# Ozonated Oil Nanoemulsion Induces Ferroptosis and Metabolic Disruption in *Candida albicans* via Membrane Destabilization

**DOI:** 10.3390/molecules31173070

**Published:** 2026-08-31

**Authors:** Ban Wang, Wenjing Fang, Hui Long, Yuxi Guo, Wenjuan Yang, Jing Wang, Nan Li, Yanni Zhao, Fuxin Chen, Pin Gong

**Affiliations:** 1 School of Food Science and Engineering, Shaanxi University of Science and Technology, Xi’an 710021, China; ban_wang@sust.edu.cn (B.W.); longhui980525@163.com (H.L.);; 2 Key Laboratory of Precision Nutrition and Functional Product Development in Xi’an, Shaanxi University of Science and Technology, Xi’an 710021, China; 3 School of Chemistry and Chemical Engineering, Xi’an University of Science and Technology, Xi’an 710054, China

**Keywords:** ferroptosis, membrane disruption, metabolic reprogramming, *Candida albicans*, vulvovaginal candidiasis

## Abstract

The poor stability and limited water dispersibility of ozonated oil constrain its antifungal applications. To overcome these limitations, we formulated an ozonated oil nanoemulsion (Nano-Ozo) and elucidated its antifungal mechanism against *Candida albicans*. Nano-Ozo was characterized by particle size, zeta potential, microstructure, and ozone retention capacity. Antifungal efficacy was assessed by minimum inhibitory concentration (MIC), membrane integrity assays, reactive oxygen species (ROS) detection, metabolomics, and a murine model of vulvovaginal candidiasis (VVC). The nanoemulsion had a uniform size (98.28 ± 5.23 nm), a high zeta potential (53.20 ± 0.28 mV), and prolonged ozone stability. It showed potent antifungal activity (MIC = 31.25 μL/mL) by disrupting membrane integrity, provoking an ROS burst, and inducing ferroptosis, as indicated by changes in SOD, MDA, LDH, and GSH levels. Metabolomic analysis revealed the inhibition of glycolysis and the TCA cycle. In the VVC model, Nano-Ozo markedly reduced vaginal fungal burden and ameliorated tissue inflammation. Given its excellent stability, biological efficacy, and multi-targeted antifungal action, Nano-Ozo holds great potential for developing localized antifungal therapies, such as topical or vaginal formulations. These findings underscore Nano-Ozo as a promising nanoengineering-based antifungal strategy.

## 1. Introduction

Ozonated oils, generated by the reaction of ozone gas with natural unsaturated oils, possess strong oxidizing and broad-spectrum antimicrobial properties [1,2]. However, their application is limited due to the instability and volatility of ozone, resulting in rapid degradation during storage and use [3]. Nanoemulsion technology offers a promising approach to overcome these limitations, by enhancing the water dispersibility, kinetic stability [4], and bioavailability of ozonated oils [5]. *Candida albicans*, a commensal fungus of human mucosal surfaces, can transform into a pathogenic organism under immunocompromised conditions [6], causing infections such as vulvovaginal candidiasis (VVC) [7]. VVC exhibits a high incidence and recurrence rate, posing significant challenges for clinical management, especially given the rise in antifungal resistance [8,9]. Therefore, the development of novel therapeutic agents with new mechanisms of action is urgently needed.

Although ozonated oil and nanoemulsions have demonstrated their own antibacterial potential [10], the method of integrating ozonated oil into stable nanoemulsions to induce ferroptosis in *Candida albicans* has not been systematically studied. Nanoemulsions, characterized by their small droplet size and large surface area [9], enhance drug penetration and antimicrobial efficiency [11,12]. Recent studies highlight that inducing ferroptosis [11]—an iron-dependent lipid peroxidation-driven form of regulated cell death—offers a novel therapeutic strategy against infections, distinct from traditional antibiotic mechanisms [13,14]. Whether the ozonated oil nanoemulsion can induce this specific form of cell death remains unclear. Likewise, the metabolic remodeling that accompanies ferroptotic death in fungi has been little explored.

To optimize a combined therapeutic strategy, we formulated a highly stable ozonated oil nanoemulsion (Nano-Ozo) and systematically assessed its antifungal activity, focusing on membrane disruption, oxidative stress, modulation of ferroptosis-related genes, and global metabolic reprogramming. We also validated its therapeutic efficacy in a murine model of VVC to provide new insights into nanoengineering-based antifungal therapy and highlight ferroptosis as a promising target for combating fungal infections.

## 2. Results

### 2.1. Nanoemulsion Formation Enhances the Stability and Dispersion of Ozonated Oil

**Figure 1:**
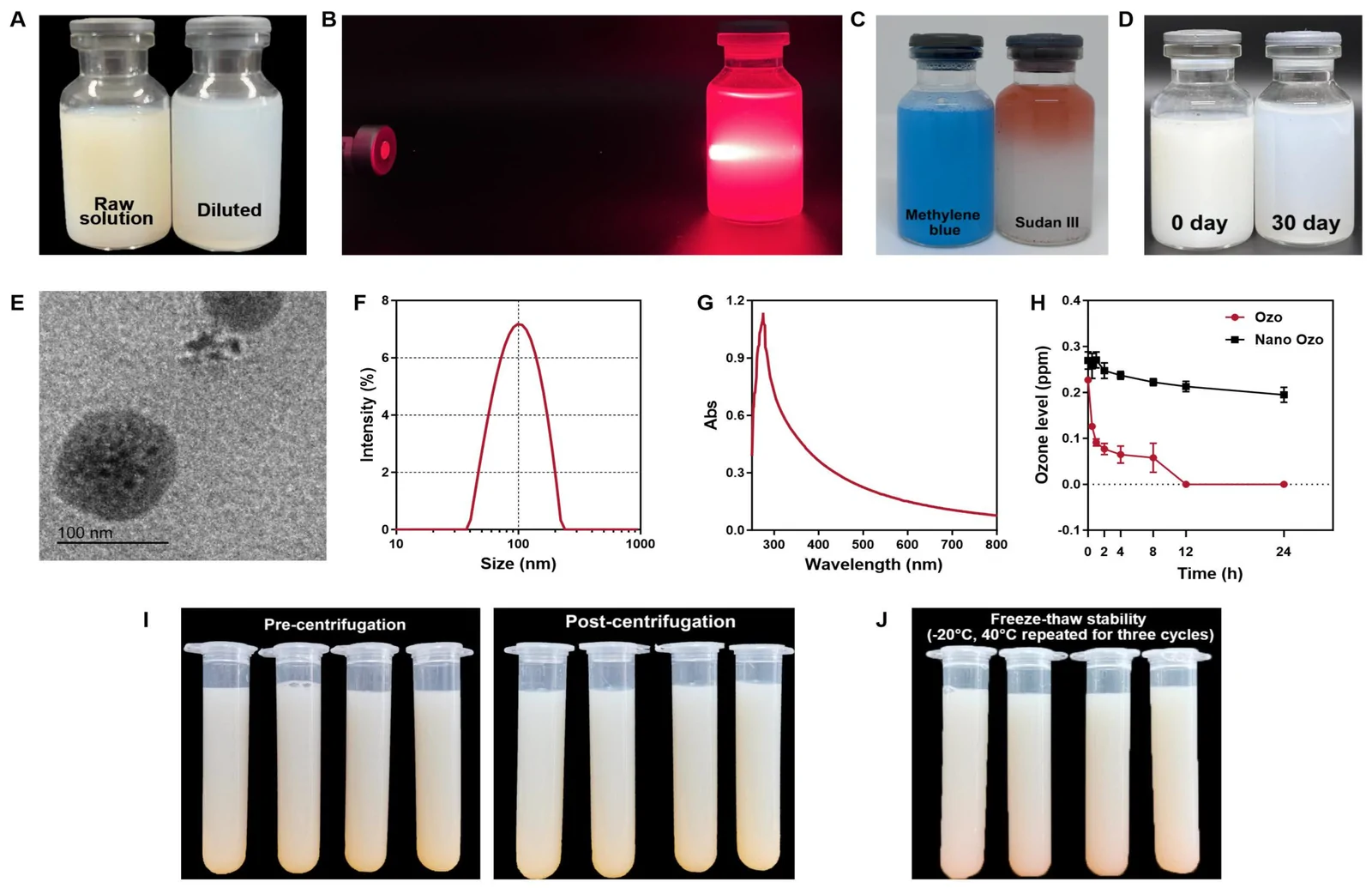
Characterization of ozonated oil nanoemulsion. (**A**) Comparison of appearance before and after dilution; (**B**) typical Tyndall effect; (**C**) identification of nanoemulsion types; (**D**) storage stability; (**E**) TEM characterization; (**F**) particle size distribution; (**G**) UV full-wavelength scanning; (**H**) changes in the ozone content of ozonated oxide oil nanoemulsions; (**I**) comparison before and after centrifugation; (**J**) freeze–thaw stability.

The prepared ozonated oil nanoemulsion exhibited a homogeneous milky appearance with no floating oil, and a light-blue opalescence was observed after dilution (Figure 1A). In contrast to the poor water solubility of raw ozonated oil, the nanoemulsions showed excellent water dispersibility. A distinct Tyndall effect confirmed the colloidal nature of the system (Figure 1B). Transmission electron microscopy revealed spherical droplets with uniform distribution and an average diameter of approximately 100 nm (Figure 1E). Dynamic light-scattering analysis showed that the nanoemulsions had a particle size of 98.28 ± 5.23 nm, a very low PDI (0.029 ± 0.002), and a high zeta potential (+53.20 ± 0.28 mV), indicating excellent uniformity and electrostatic stability (Figure 1F). The pH values ranged from 6.5 to 7.2, suitable for biological applications. Type-identification tests using Sudan III and methylene blue indicated that the system was an oil-in-water (O/W) nanoemulsion (Figure 1C). UV spectroscopy showed a characteristic absorption peak at 275 nm (Figure 1G). Ozone content measurements demonstrated that free ozonated oil degraded rapidly within 2 h, whereas in the nanoemulsion form, ozone release was significantly prolonged, maintaining about 72% of initial content after 24 h (Figure 1H). Centrifugation, freeze–thaw cycling, and one-month room temperature storage tests showed no phase separation or precipitation (Figure 1D,I), confirming the outstanding physical and chemical stability of the nanoemulsion.

### 2.2. Ozonated Oil Nanoemulsions Effectively Inhibit the Growth and Morphology of Candida albicans

**Figure 2:**
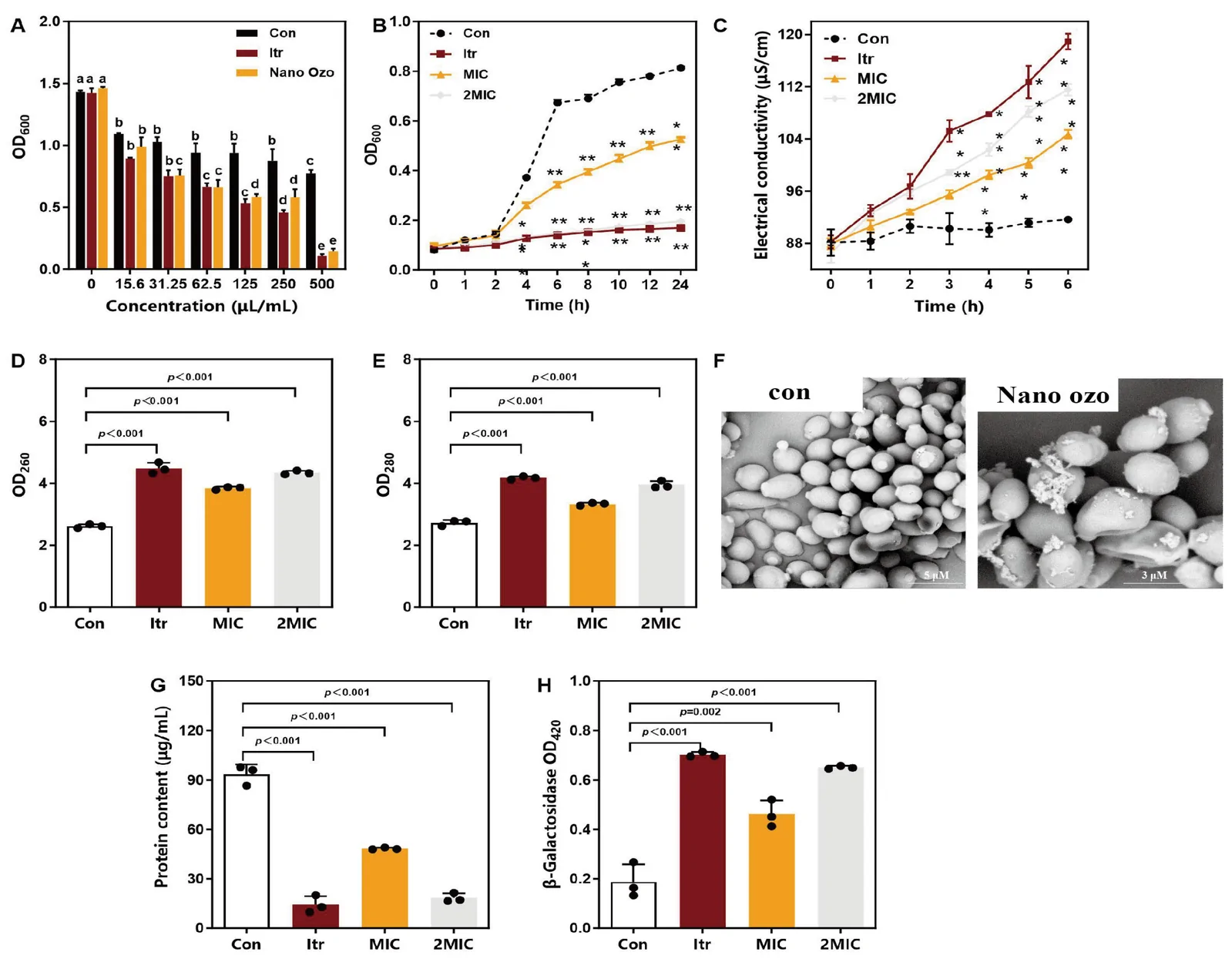
Inhibitory activity of ozonated oil nanoemulsion against *Candida albicans*. (**A**) MIC; (**B**) inhibition growth curve; (**C**) conductivity; (**D**) DNA content (OD_260_); (**E**) RNA content (OD_280_); (**F**) SEM characterization (12,000- and 21,500-fold magnifications); (**G**) protein content; (**H**) relative characterization of β-galactosidase OD_420_. Con, untreated control group; data are presented as mean ± SD (*n* = 3). Statistical significance: compared with the Con group, * *p* < 0.05, ** *p* < 0.01.

The minimum inhibitory concentration (MIC) of the ozonated oil nanoemulsion against *Candida albicans* was determined to be 31.25 μL/mL (Figure 2A). Although the blank nanoemulsion showed mild antibacterial activity, likely due to isopropyl myristate, the ozonated oil nanoemulsion exhibited substantially stronger inhibition, comparable to the positive control itraconazole, indicating that the antifungal effect primarily derives from the ozonated oil. Growth curve analysis (Figure 2B) showed that treatment at the MIC significantly lengthened the lag phase and reduced the logarithmic growth rate relative to the control, while the 2×MIC almost completely suppressed proliferation over 24 h observation, keeping OD_600_ near baseline. Scanning electron microscopy (Figure 2F) revealed that untreated *Candida albicans* cells had smooth, round, intact surfaces, whereas Nano-Ozo-treated cells displayed surface wrinkling, shrinkage, collapse, and marked cytoplasmic leakage, findings that align with biochemical evidence of membrane disruption. Together, these results indicate that Nano-Ozo exerts potent, concentration-dependent antifungal activity primarily by disrupting outer and inner membrane structures.

### 2.3. Ozonated Oil Nanoemulsions Induce Membrane Damage and Oxidative Stress in Candida albicans

Treatment with Nano-Ozo significantly increased the electrical conductivity of *Candida albicans* suspensions, reflecting enhanced membrane permeability and leakage of intracellular ions (Figure 2C). Simultaneously, OD260 and OD280 measurements revealed substantial nucleic acid leakage following treatment with MIC and 2×MIC of Nano-Ozo, comparable to itraconazole (Figure 2D,E). Protein leakage assays demonstrated a marked decrease in intracellular protein content after Nano-Ozo exposure, further confirming membrane disruption (Figure 2G). The ONPG hydrolysis assay showed a concentration-dependent increase in OD420, indicating increased inner membrane permeability (Figure 2H).

**Figure 3:**
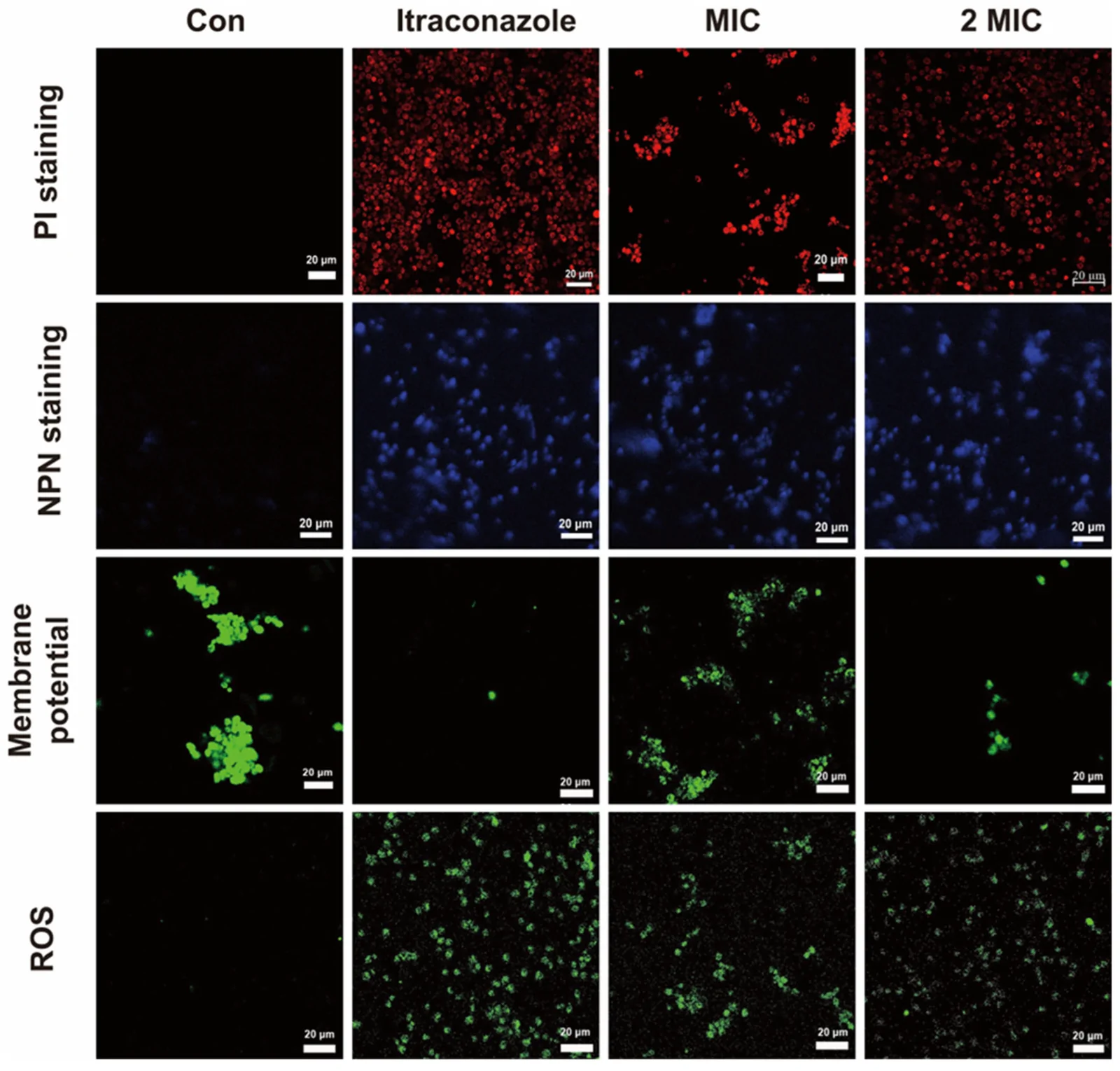
Effect of ozonated oil nanoemulsion on the *Candida albicans* cell membrane. PI staining: red fluorescence; NPN staining: blue fluorescence; Rhodamine 123 staining: yellow-green fluorescence; DCFH-DA staining: green fluorescence (Con, untreated control group).

Fluorescence staining with propidium iodide (PI) revealed significant red fluorescence in treated groups, implying a loss of membrane integrity and DNA binding by PI (Figure 3). Enhanced NPN uptake also confirmed damage to the outer membrane of *Candida albicans*. Rhodamine 123 assays indicated a sharp decrease in mitochondrial membrane potential after Nano-Ozo treatment, demonstrating early signs of apoptosis or necrosis. Furthermore, DCFH-DA staining revealed a strong green fluorescence signal, suggesting excessive intracellular ROS accumulation, which intensified with increasing Nano-Ozo concentration (Figure 3). These results collectively demonstrate that Nano-Ozo disrupts the membrane structure of *Candida albicans*, induces oxidative stress, and ultimately promotes fungal cell death.

### 2.4. Nano-Ozo Disrupts Amino Acid and Central Carbon Metabolism in Candida albicans

**Figure 4:**
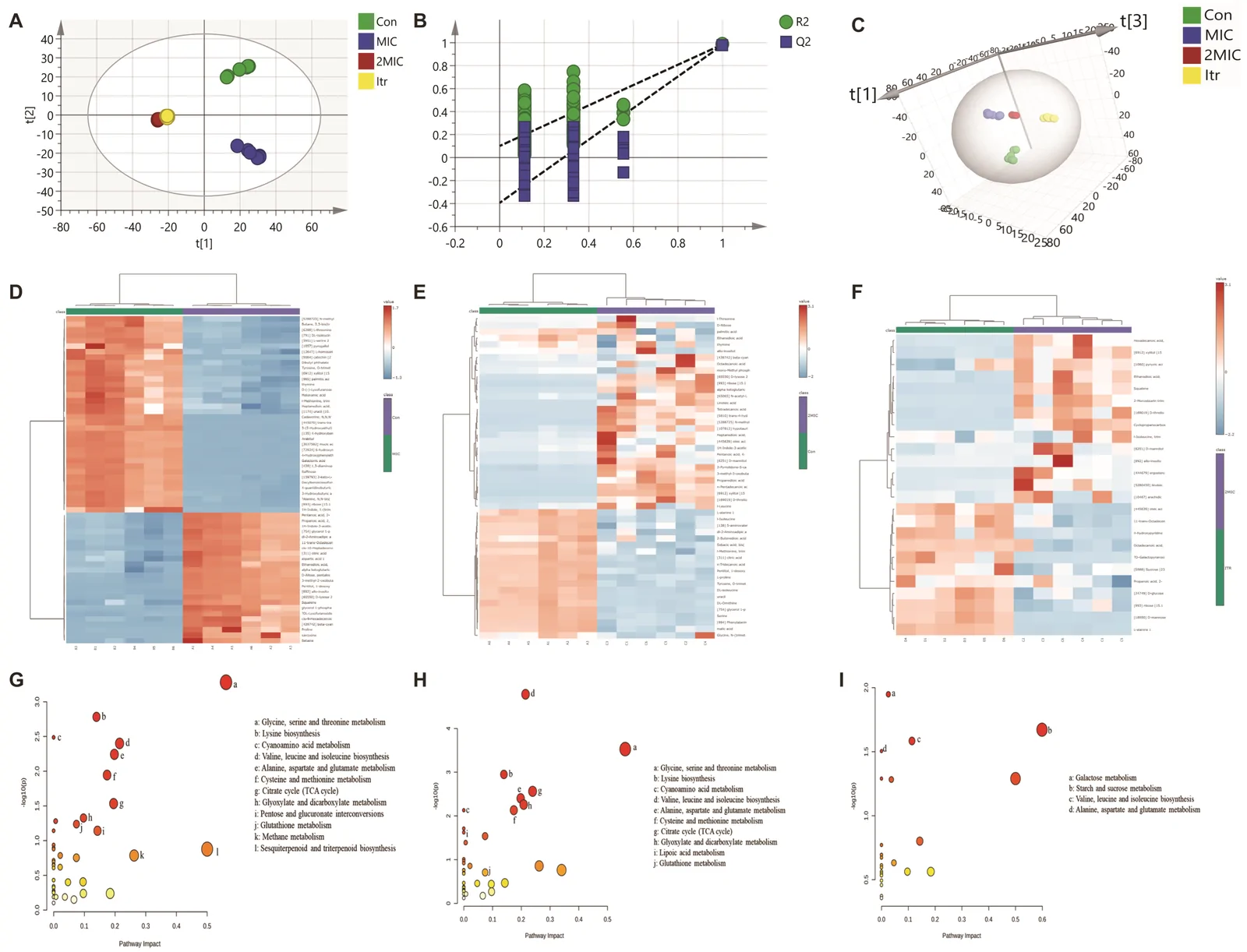
Metabolomic analysis of the inhibitory effect of Nano-Ozo on *Candida albicans*. (**A**) PLS-DA score of metabolites; (**B**) permutation test analysis of the PLS-DA model; (**C**) 3D figure of PLS-DA; (**D**–**F**) heat maps of different metabolites for Con and MIC groups, Con and 2×MIC groups, Itr and 2×MIC groups; (**G**–**I**) maps of differential metabolite metabolic pathways for Con and MIC groups, Con and 2×MIC groups, Itr and 2×MIC groups.

Untargeted GC-MS metabolomics analysis revealed significant metabolic perturbations in *Candida albicans* following Nano-Ozo treatment at MIC and 2×MIC. PCA and OPLS-DA analyses demonstrated clear separation between treated and control groups, confirming extensive metabolic reprogramming (Figure 4A–C). At the MIC level, Nano-Ozo increased levels of N-methyl-D-alanine, L-threonine, and xylitol, while reducing proline, sarcosine, and glycerophosphates. Pathway enrichment analysis indicated the disruption of glycine, serine, threonine metabolism, branched-chain amino acid biosynthesis, and glycolysis (Figure 4G). Higher Nano-Ozo concentrations (2×MIC) further impaired metabolic pathways, notably the TCA cycle, glutathione metabolism, and fatty acid biosynthesis (Figure 4H). Decreases in citric acid and α-ketoglutarate alongside the accumulation of L-arabinose and pentadecanoic acid suggested the suppression of energy metabolism and redox imbalance. Compared with itraconazole, Nano-Ozo induced similar metabolic changes, particularly targeting glycolysis and amino acid metabolism (Figure 4I). Overall, Nano-Ozo exerts antifungal effects by disrupting critical biosynthetic and energy-generating pathways in *Candida albicans*, leading to metabolic stress and cellular dysfunction.

### 2.5. Nano-Ozo Ameliorates Vaginal Inflammation and Reduces Fungal Burden in VVC Mice

**Figure 5:**
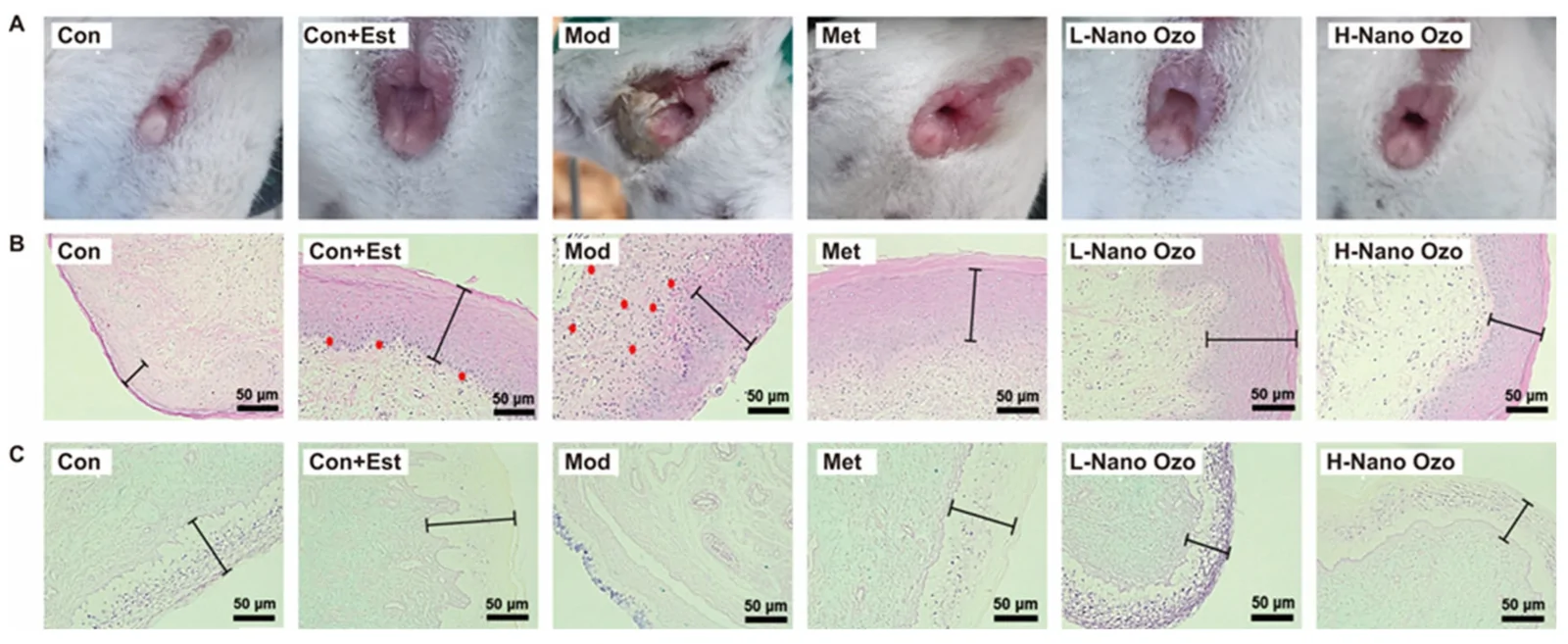
Effect of Nano Ozo on VVC vaginal tissue. (**A**) Vaginal appearance of different treated VVC mice; (**B**) HE staining of mouse vaginal tissue (200-fold); (**C**) PAS staining of mouse vaginal tissue (200-fold). (Con, untreated control group).

Nano-Ozo treatment significantly alleviated vaginal inflammation symptoms in vulvovaginal candidiasis (VVC) mice (Figure 5). The model group exhibited severe swelling, ulceration, and increased vaginal discharge, whereas mice treated with low- and high-dose Nano-Ozo or diazoxide vaginal gel showed markedly reduced redness, swelling, and secretions. Histological analysis (HE and PAS staining) further revealed that Nano-Ozo restored vaginal tissue morphology, reduced epithelial hyperkeratosis, and minimized inflammatory cell infiltration, particularly at high concentrations. Quantification of *Candida albicans* load from vaginal lavage samples demonstrated that Nano-Ozo substantially suppressed fungal colonization (Table 1). After 20 days of treatment, the fungal load in the high-dose Nano-Ozo group decreased from (921 ± 103) × 10 CFU/mL to (13 ± 4) × 10 CFU/mL, comparable to the reduction achieved by diazoxide vaginal gel. These results confirm that Nano-Ozo effectively mitigates VVC symptoms and reduces *Candida albicans* burden in vivo, supporting its potential as a therapeutic agent for fungal vaginitis.

### 2.6. Nano-Ozo Downregulates Pro-Inflammatory Cytokines in VVC Mice

**Figure 6:**
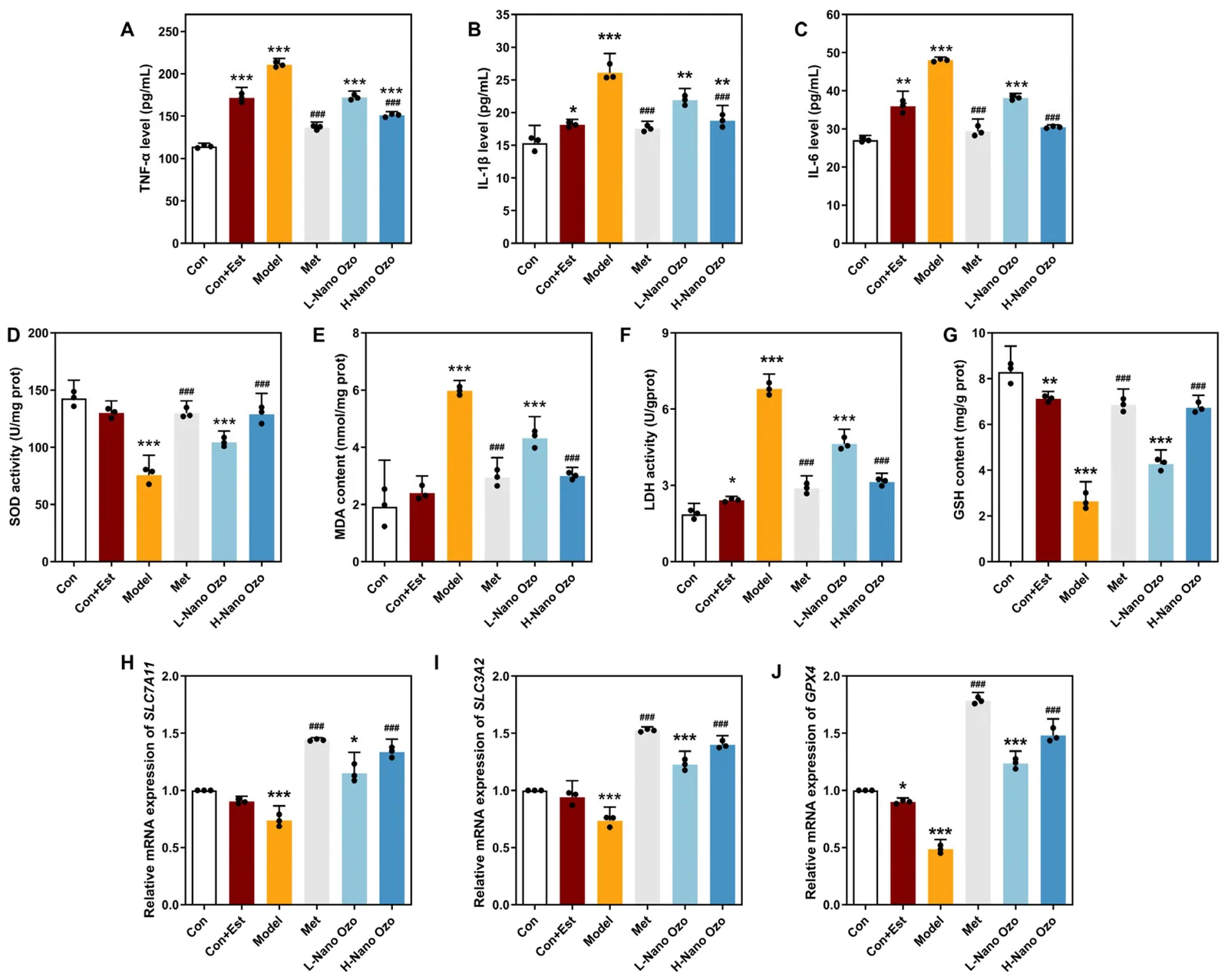
Characterization of serum inflammatory factor levels, ferroptosis-related indices, and gene expression in VVC mice. (**A**–**C**) TNF-α, IL-1β, and IL-6 levels; (**D**) SOD activity; (**E**) MDA; (**F**) LDH activity; (**G**) GSH content; (**H**–**J**) relative mRNA expression of *SLC7A11*, *SLC3A2*, and *GPX4*. Con, untreated control group; data are presented as mean ± SD (*n* = 3). * Denotes analysis of significance compared to Con (* *p* < 0.05, ** *p* < 0.01, *** *p* < 0.01); # denotes analysis of significance compared to Mod (^###^ *p* < 0.01).

Nano-Ozo treatment effectively modulated the serum levels of key pro-inflammatory cytokines in VVC mice (Figure 6A–C). Compared to the control group, the model group exhibited significantly elevated levels of TNF-α, IL-1β, and IL-6 (*p* < 0.01). Low-dose Nano-Ozo treatment did not substantially alleviate this inflammatory surge, whereas high-dose Nano-Ozo and diazoxide vaginal gel significantly reduced the serum concentrations of TNF-α, IL-1β, and IL-6 compared to the model group (*p* < 0.01). These findings suggest that Nano-Ozo, particularly at high concentrations, can effectively suppress systemic inflammatory responses in VVC mice, achieving therapeutic efficacy comparable to that of the clinically used diazoxide vaginal gel.

### 2.7. Nano-Ozo Attenuates Oxidative Damage and Ferroptosis Markers in Vaginal Tissues of VVC Mice

Ferroptosis, a type of regulated cell death caused by iron overload and lipid peroxidation, can be evaluated through oxidative stress markers and ferroptosis-related genes. Increased oxidative stress depletes intracellular glutathione (GSH), leading to GPX4 inactivation, elevated malondialdehyde (MDA) and lactate dehydrogenase (LDH) levels, and reduced superoxide dismutase (SOD) activity. As shown in Figure 6D, SOD activity in the vaginal tissues of the model (Mod) group is significantly lower than that in the control (Con) group (75.63 U/mg vs. control, *p* < 0.01). After treatment with low-dose and high-dose Nano-Ozo, SOD activity significantly increased to 104.4 U/mg and 128.85 U/mg, respectively (*p* < 0.01 vs. Mod group), with high-dose Nano-Ozo restoring SOD activity close to normal levels.

MDA and LDH levels, indicators of lipid peroxidation and membrane rupture, were significantly elevated in the Mod group compared to controls (*p* < 0.01) (Figure 6E,F). Nano-Ozo treatment, at both low and high concentrations, markedly reduced MDA and LDH levels (*p* < 0.01 vs. Mod group), with the high-dose group showing effects comparable to the positive-control diazoxide group. In addition, GSH levels, critical for antioxidant defense, were substantially decreased in the Mod group (2.64 mg/g prot, *p* < 0.01 vs. Con group) (Figure 6G). Treatment with low-dose and high-dose Nano-Ozo increased GSH content to 4.26 mg/g prot and 6.73 mg/g prot, respectively (*p* < 0.01 vs. Mod group), with the high-dose group showing no significant difference from the positive control. Collectively, these biochemical results demonstrate that Nano-Ozo attenuates vaginal tissue inflammation by mitigating oxidative stress and inhibiting ferroptosis pathways.

### 2.8. Nano-Ozo Upregulates SLC7A11, SLC3A2, and GPX4 to Inhibit Ferroptosis in Vaginal Tissues of VVC Mice

The regulation of ferroptosis is closely related to the cystine/glutamate antiporter System XC^−^ (composed of SLC3A2 and SLC7A11) and the antioxidant enzyme GPX4. Downregulation of SLC7A11 impairs cystine transport, depleting GSH levels, leading to GPX4 inactivation and triggering lipid peroxidation and ferroptotic cell death [15,16]. As shown in Figure 6H, the expression of *SLC7A11* in the vaginal tissues of the Mod group was reduced by 0.74-fold compared to the Con group (*p* < 0.01). After Nano-Ozo treatment, *SLC7A11* expression increased by 1.14-fold in the L-Nano Ozo group (*p* < 0.01), 1.34-fold in the H-Nano Ozo group (*p* < 0.01), and 1.43-fold in the Met group (*p* < 0.01), compared to the Mod group. There was no significant difference between the H-Nano Ozo group and the Met group, suggesting that Nano-Ozo effectively restores SLC7A11 expression. Similarly, *SLC3A2* expression was significantly reduced by 0.8-fold in the Mod group compared to the Con group (*p* < 0.01) (Figure 6I). Treatment with Nano-Ozo increased *SLC3A2* expression by 1.24-fold in the L-Nano Ozo group (*p* < 0.01) and 1.42-fold in the H-Nano Ozo group (*p* < 0.001), with no significant difference from the Met group (1.52-fold increase, *p* < 0.01). Regarding *GPX4*, a critical enzyme for eliminating lipid peroxides, its expression was decreased by 0.485-fold in the Mod group compared to the Con group (*p* < 0.01) (Figure 6J). After Nano-Ozo treatment, *GPX4* expression increased by 1.23-fold in the L-Nano Ozo group and 1.48-fold in the H-Nano Ozo group (*p* < 0.01 vs. Mod group). Overall, these results suggest that VVC induces ferroptosis by suppressing *SLC7A11*, *SLC3A2*, and *GPX4* expression, while Nano-Ozo treatment reverses these effects, thus inhibiting ferroptosis and contributing to the therapeutic effects against Candida vaginitis.

## 3. Discussion

The present study systematically demonstrates that ozonated oil nanoemulsions (Nano-Ozo) exhibit potent antifungal effects against *Candida albicans* through a multifactorial mechanism involving membrane disruption, ferroptosis induction, and metabolic reprogramming. The nanoemulsions were characterized by stable colloidal dispersion, narrow particle size distribution, and a favorable zeta potential, contributing to excellent physical stability and prolonged ozone retention over storage, thereby meeting the fundamental requirements for practical therapeutic applications. Nano-Ozo exerted strong inhibitory effects against *C. albicans* with a minimum inhibitory concentration (MIC) of 31.25 μL/mL. Growth curve analysis revealed that Nano-Ozo significantly prolonged the lag phase and suppressed the logarithmic growth phase, ultimately preventing cell proliferation. Notably, the antifungal efficacy of Nano-Ozo was comparable to itraconazole, suggesting its potential as an alternative or adjunctive antifungal therapy.

Mechanistic studies showed that Nano-Ozo caused pronounced damage to the fungal cell membrane, as evidenced by increased membrane permeability [17], electrical conductivity, and leakage of nucleic acids and proteins [18,19]. The enhanced uptake of propidium iodide and NPN fluorescence further confirmed severe membrane disruption [20,21]. In parallel, Nano-Ozo induced substantial oxidative stress within *C. albicans* cells, leading to elevated intracellular ROS accumulation, membrane depolarization, and mitochondrial dysfunction [22,23]. Importantly, this study implicates ferroptosis as a critical pathway underlying Nano-Ozo-induced fungal cell death. Ferroptosis is characterized by iron-dependent lipid peroxidation and oxidative collapse [20,21]. Nano-Ozo treatment significantly elevated malondialdehyde (MDA) and lactate dehydrogenase (LDH) levels while decreasing superoxide dismutase (SOD) activity and glutathione (GSH) content in *C. albicans* cells, collectively indicating oxidative membrane damage. Concurrently, Nano-Ozo upregulated the expression of key ferroptosis-regulatory genes, including *SLC7A11* and *GPX4* [24,25]. SLC7A11 promotes cystine uptake for GSH synthesis, while GPX4 detoxifies lipid peroxides [15]. Upregulation of these genes suggests a compensatory antioxidant response attempting to mitigate ferroptotic damage.

Furthermore, different from the previously reported liposomal ozonated oil preparations that mainly act through membrane oxidation [26], our study reveals a dual mechanism involving direct membrane disruption and the metabolism–ferroptosis cascade. The metabolomic analysis reinforced the ferroptosis-related findings by unveiling profound disruptions in amino acid metabolism, glycolysis, and the TCA cycle. Specifically, the depletion of proline and α-ketoglutarate, and the accumulation of N-methyl-D-alanine, reflected an imbalance in cellular redox homeostasis and impaired energy metabolism, both recognized hallmarks of ferroptotic progression [27,28,29]. Proline and α-ketoglutarate are closely involved in maintaining mitochondrial function and ROS scavenging. These metabolites are crucial for NADPH regeneration and glutathione synthesis. Their reduction impairs the antioxidant capacity of cells, making Staphylococcus albus prone to lethal lipid peroxidation. Thus, their depletion under Nano-Ozo treatment likely exacerbated oxidative stress and promoted lipid peroxidation [30].The inhibition of glycolytic flux and TCA cycle intermediates further deprived *C. albicans* of essential energy sources, compounding ferroptosis-mediated cell death [29,31]. This metabolic sensitization explains why even sub-inhibitory concentrations of Nano-Ozo can effectively trigger ferroptosis in fungal cells. In the in vivo murine model of vulvovaginal candidiasis (VVC), Nano-Ozo treatment significantly alleviated vaginal symptoms, reduced fungal burden, and restored tissue integrity. Moreover, Nano-Ozo markedly suppressed systemic inflammatory cytokines (TNF-α, IL-1β, and IL-6), reduced oxidative stress markers (MDA and LDH), and enhanced antioxidant defenses (SOD and GSH) in vaginal tissues. Notably, Nano-Ozo also upregulated ferroptosis-inhibitory genes (*SLC7A11*, *SLC3A2*, *GPX4*) in vaginal tissues, further corroborating the in vitro findings. These integrative findings establish that Nano-Ozo exerts antifungal effects by simultaneously compromising membrane integrity, collapsing metabolic networks, and promoting ferroptosis, thereby achieving synergistic cellular lethality against *Candida albicans*.

Nevertheless, the underlying molecular mechanisms by which Nano-Ozo precisely regulates ferroptosis-related signaling pathways and modulates host–pathogen interactions warrant further elucidation. In addition, while our stability assessments were supported by photographic documentation and initial DLS characterization, quantitative DLS measurements after centrifugation, freeze–thaw cycles, and long-term storage were not performed in the current study. Future studies should focus on optimizing Nano-Ozo formulations for targeted delivery, evaluating long-term safety profiles, and exploring clinical translation, especially for localized antifungal therapies such as vaginal gels, sprays, or implants.

## 4. Materials and Methods

### 4.1. Materials

Soy lecithin, polyethylene glycol-400, 1,2-propanediol, Tween-80, and Spectra-80 were obtained from Tianjin Tianli Chemical Reagent Co., Ltd. (Tianjin, China). Isopropyl myristate, methylene blue, and BCA kit were purchased from Shanghai Yuanye Biotechnology Co., Ltd. (Shanghai, China). Itraconazole was supplied by Xi’an Janssen Pharmaceutical Co., Ltd. (Xi’an, China). Other reagents were of analytical grade. *Candida albicans* was provided by Associate Professor Qian Weidong, School of Food Science and Engineering, Shaanxi University of Science and Technology.

### 4.2. Preparation of Ozonated Oil Nanoemulsions

Nanoemulsions were prepared by ultrasound-assisted emulsification. An oil phase comprising isopropyl myristate (3.5%) and ozonated oil (1.0%) was mixed with Tween-80 (22%) and polyethylene glycol-400 (7%) under optimized conditions. The mixture was subjected to ultrasonication (300 W, 24 min) to form stable nanoemulsions.

### 4.3. Characterization of Nanoemulsions

Particle size, polydispersity index (PDI) [32], and zeta potential [33] were measured using a nanoparticle size analyzer (Malvern Panalytical Ltd., Worcestershire, UK). Morphology was observed by transmission electron microscopy after negative staining [34]. Nanoemulsion type was determined using Sudan III and methylene blue staining methods [35,36]. UV-visible spectroscopy was employed to measure absorption peaks [37]. Stability was evaluated by centrifugation, freeze–thaw cycling, and storage tests [38].

### 4.4. Antifungal Activity Assays

The minimum inhibitory concentration (MIC) was determined by the twofold dilution method in 96-well plates [39,40]. Growth curves were plotted by measuring OD600 at different time points. Changes in cell morphology were observed by scanning electron microscopy (SEM). Membrane integrity was assessed through conductivity measurement [41], nucleic acid/protein leakage assays [42], propidium iodide (PI) staining [43], NPN uptake [44], and ONPG hydrolysis [45]. Membrane potential changes were evaluated by Rhodamine 123 fluorescence [45,46], and intracellular reactive oxygen species (ROS) accumulation was detected using DCFH-DA [47].

### 4.5. Metabolomic Analysis

Treated *Candida albicans* cells were subjected to metabolite extraction using 80% methanol containing internal standards. After derivatization with methoxyamine hydrochloride and MSTFA, samples were analyzed by GC-MS. Data were processed using SIMCA 14.1 software for OPLS-DA modeling [48]. Differential metabolites were screened based on VIP > 1 and *p* < 0.05, and pathway enrichment was conducted via MetaboAnalyst 6.0.

### 4.6. Animal Study

Female Kunming mice were used to establish a vulvovaginal candidiasis (VVC) model via estradiol benzoate induction followed by vaginal inoculation with *Candida albicans* [49]. Mice were treated with low-dose (1%) or high-dose (3%) Nano-Ozo, or with diazoxide vaginal gel as the positive control. Vaginal lavage samples were collected to assess fungal load. Vaginal tissues were subjected to histological analysis (HE and PAS staining) and detection of inflammatory cytokinesn (IL-6, IL-1β, TNF-α) and ferroptosis markers (SOD, GSH, MDA, LDH).

### 4.7. qRT-PCR Assays

The primer design process was mainly conducted according to Table S1. The total RNA of vaginal tissues was extracted using the Total RNA Extractor kit, and the RNA content was measured with the Nano Drop (Thermo Fisher Scientific, Waltham, MA, USA). After quantification, reverse transcription was performed. The expression levels of ferroptosis-related genes (SLC7A11, SLC3A2, GPX4) were quantified using qRT-PCR and normalized to GAPDH. All statistical values are presented as 2^−ΔΔCt^.

### 4.8. Statistical Analysis

All data are expressed as mean ± standard deviation (SD). One-way ANOVA followed by Dunnett’s multiple comparisons test was performed using GraphPad Prism 8.0. A *p*-value < 0.05 was considered statistically significant.

## 5. Conclusions

In this study, we developed a stable and bioactive ozonated oil nanoemulsion (Nano-Ozo) that demonstrated potent antifungal activity against *Candida albicans* through membrane disruption, ferroptosis induction, and metabolic reprogramming. Nano-Ozo effectively impaired fungal membrane integrity, triggered oxidative stress, and disrupted key metabolic pathways, ultimately leading to ferroptotic cell death. Moreover, Nano-Ozo significantly alleviated vaginal inflammation, reduced fungal burden, and regulated ferroptosis-associated biomarkers and genes in a vulvovaginal candidiasis (VVC) mouse model. These findings highlight the therapeutic potential of Nano-Ozo as a novel antifungal agent with a dual-action mechanism, offering a promising alternative for the prevention and treatment of fungal infections. Future studies will focus on optimizing the formulation, elucidating long-term safety, and exploring the clinical translation of Nano-Ozo-based antifungal therapies.

## Figures and Tables

**Table 1:** Changes in *Candida albicans* load in the vaginas of mice (unit: ×10 CFU/mL).

Group	Pre-Dose	Dose 5 Days	Dose 10 Days	Dose 15 Days	Dose 20 Days
Con	-	-	-	-	-
Con + Est	-	-	-	-	-
Mod	983 ± 176	995 ± 134	1089 ± 204	1157 ± 1 28	1265 ± 145
Met	936 ± 154	578 ± 101	218 ± 67 *	89 ± 23 **	8 ± 5 *
L-Nano ozo	895 ± 112	635 ± 121	329 ± 78 *	176 ± 45 **	67 ± 12 *
H-Nano ozo	921 ± 103	569 ± 124	234 ± 47 *	105 ± 34 **	13 ± 4 *

Note: *Candida albicans* vaginal load data are presented as mean ± SD (*n* = 3). Compared with the Mod group, * *p* < 0.05 and ** *p* < 0.01.

## Data Availability

The data that support the findings of this study are available from the corresponding authors upon reasonable request.

## Supplementary Materials

The following supporting information can be downloaded at: https://www.mdpi.com/article/10.3390/molecules31173070/s1, Table S1. Primer sequences; Table S2. Differential metabolites between Con group and 2×MIC group.
