# Supplementary material for: Ozonated Oil Nanoemulsion Induces Ferroptosis and Metabolic Disruption in Candida albicans via Membrane Destabilization
Source: Molecules. 2026 Aug 31;31(17):3070. doi: 10.3390/molecules31173070 (PMC13567357; doi:10.3390/molecules31173070)
Supplement: Supplementary file 1 [file molecules-31-03070-s001.zip › molecules-4432139-supplementary.pdf]

Supplementary materials

Table S1. Primer Sequences

Gene	NCBI Gene ID	Primer sequence	Number of bases
GAPDH(F)	NM_001289726.1	TGGTATCGTGGAAGGACTC	19
GAPDH(R)		AGTAGAGGCAGGGATGAGT	19
SLC7A11(F)	NM_011990.2	TGCTGGGCTGATTTTATCTTCG	22
SLC7A11(R)		GAAAGGGCAACCATGAAGAGG	21
SLC3A2(F)	NM_001161413.1	CTGGTGCCGTGGTCATAATC	20
SLC3A2(R)		GCTCAGGTAATCGAGACGCC	20
GPX4(F)	NM_008162.3	GCAACCAGTTTGGGAGGCAG	20
GPX4(R)		CCTCCATGGGACCATAGCGC	20

Table S2. Differential Metabolites between Con Group and 2×MIC Group

No.	Metabolite name	Database number	VIP values
1	L-Proline	HMDB0000162	7.20874
2	L-Lsoleucine	HMDB0000172	6.92486
3	L-Valine	HMDB0000883	5.88743
4	5-Aminovaleric acid	HMDB0003355	5.87632
5	Norvaline	HMDB0013716	5.87479
6	Palmitic acid	HMDB0000220	5.6767
7	Ethanedioic acid	HMDB0002329	5.42576
8	Aspartic acid	HMDB0000191	5.04215
9	L-Glutamic acid	HMDB0000148	4.991
10	Yracil	HMDB0000300	4.93675
11	DL-Isoleucine	HMDB0000172	4.74092
12	Hexadecanoic acid	HMDB0000220	4.44296
13	Propanedioic acid	HMDB0000691	4.4035
14	Phenylalanine	HMDB0000159	4.37211
15	Octadecanoic acid	HMDB0000827	4.29108
16	L-Threonine	HMDB0000167	4.19064
17	Serine	HMDB0000187	4.14477
18	L-Leucine	HMDB0000687	3.99702
19	Tridecanoic acid	HMDB0000910	3.81832
20	L-Tyrosine	HMDB0000158	3.6267
21	L-Alanine	HMDB0000161	3.55109
22	Citric acid	HMDB0000094	3.44893
23	D-Xylitol	HMDB0002917	3.35904
24	Valeric acid	HMDB0000892	3.35555
25	Cis-9-Hexadecenoic acid	HMDB0003229	3.21185
26	Glycerol 3-phosphate	HMDB0000126	3.06534
27	Pyroglutamic acid	HMDB0000267	3.01836
28	D-Ribose	HMDB0000283	2.74401
29	Pyruvic acid	HMDB0000243	2.58672
30	Oleic acid	HMDB0000207	2.5043
31	Methionine	HMDB0000696	2.36712
32	Tetradecanoic acid	HMDB0000806	2.33957
33	Linoleic acid	HMDB0000673	2.27733
34	Sebacic acid	HMDB0000792	1.97853
35	Glycine	HMDB0000123	1.95544
36	Alpha ketoglutaric acid	HMDB0000208	1.9079
37	L-Homoserine	HMDB0000719	1.76914
38	N-acetyl-L-aspartic acid	HMDB0000812	1.74089
39	Pentitol	HMDB0000508	1.62354
40	1H-Indole-3-acetic acid	HMDB0000197	1.57175
41	Pentadecanoic acid	HMDB0000826	1.54368
42	N-Methylalanine	HMDB0094692	1.47067
43	4-Hydroxyproline	HMDB0000725	1.40448
44	Aminoadipic acid	HMDB0000510	1.30362
45	L-Aspartic acid	HMDB0000191	1.29936
46	Mono-Methyl phosphate	HMDB0061711	1.19511
47	Thymine	HMDB0000262	1.18116

48	Pyrophosphate	HMDB0000250	1.17059
49	D-Ribose	HMDB0000283	1.16021
50	2-Butenedioic acid	HMDB0000176	1.15835
51	Hypotaurine	HMDB0000965	1.15217
52	Betaine	HMDB0000043	1.08984
53	D-Threitol	HMDB0004136	1.0662
54	Malic acid	HMDB0000156	1.05562
55	3-Methyl-2-Oxobutanoic acid	HMDB0000019	1.04675
56	Pimelic acid	HMDB0000857	1.03294
57	Mannitol	HMDB0000765	1.02968
